# The impact of social support for older adults in nursing homes on successful aging: a moderated mediation model

**DOI:** 10.3389/fpubh.2024.1351953

**Published:** 2024-02-21

**Authors:** Fan Xu, Jiquan Zhang, Shaoju Xie, Qiao Li, Yaoyao Long

**Affiliations:** ^1^Oncology Department, Deyang People's Hospital, Deyang, Sichuan, China; ^2^Nephrology Department, Deyang People's Hospital, Deyang, Sichuan, China

**Keywords:** nursing homes, older adults, social support, meaning in life, frailty, successful aging, moderating mediation

## Abstract

**Objective:**

To investigate the connection between social support (SS) and successful aging (SA) in older adults residing in nursing homes, examining the mediating role of meaning in life (MIL). Additionally, this study aims to assess whether frailty moderates the mediation model.

**Methods:**

A cross-sectional survey approach was employed to recruit older adults from six nursing homes in Sichuan Province between August 2022 and December 2022. Questionnaires, including the General Information Questionnaire, Social Support Rating Scale (SSRS), Meaning in Life Questionnaire (MLQ), Tilburg Frailty Indicator (TFI), and Successful Aging Inventory (SAI), were administered. Data obtained from the completed questionnaires were analyzed using SPSS and its macro program PROCESS.

**Results:**

SS emerged as a noteworthy positive predictor of SA in older adults of nursing homes. MIL was identified as a partial mediator in the link between SS and SA. Furthermore, frailty attenuated the positive predictive impact of MIL on SA and moderated the latter part of the mediation model, wherein SS influences SA through MIL. The influence of MIL on SA was more pronounced in older adults with lower frailty levels in nursing homes, while it was diminished in those with higher levels of frailty.

**Conclusion:**

Apart from ensuring the availability of essential medical resources in long-term care for older adults, workers in nursing homes should also recognize the significance of “spiritual aging” to cultivate a sense of MIL among older adults. Simultaneously, attention must be directed toward screening for frailty indicators in older adults. Psychological care and physical exercise programs should be intensified for older adults with a high level of frailty, aiming to decelerate the progression of frailty in nursing home residents. This approach leverages the mediating role of MIL and the moderating influence of frailty, ultimately enhancing SA and promoting healthy aging in older adults within nursing home settings.

## 1 Introduction

Global population aging poses a significant challenge, and the aging situation in China has intensified. As of the end of 2021, data from the 2021 National Bulletin on the Development of the Aging Career (1) revealed that there were 267.36 million persons 60 year and older, constituting 18.9% of the total population. Additionally, there were 2,005.06 million persons 65 year and older, making up 14.2% of the total population. It is projected that by ~2,035, the number of individuals aged 60 and over will surpass 400 million, marking a substantial aging phase. Despite being in the primary stage of socialism, China's social services structure remains imperfect. The current services for the aging inadequately address the challenges posed by population aging, such as diminishing labor supply, economic growth reduction, and weakened consumer demand. Consequently, addressing aging-related issues has become a critical area of exploration, with a focus on the concept of Successful Aging (SA) and its associated practices.

SA serves as a crucial indicator of the health status among the older population (2). According to the model presented by Rowe, achieving SA necessitates older adults to fulfill three key criteria: maintaining little to no risk of disease and disability; exhibiting elevated levels of physical function and cognitive ability; and engaging in sustained and active participation in social activities (3). However, the inclusion of the “no risk of disease and disability” factor in the model may exclude certain older adults who consider themselves successful in old age despite having chronic diseases. Consequently, an increasing body of research argues that the study of SA should not only concentrate on the biological dimension but should also delve into the psychological and social dimensions. In 2005, Flood, an American nursing expert, introduced the mid-range nursing theory of SA through a systematic analysis of literature, integrating Roy's adaptive model with the social theory of transcending aging (4). According to this theory, SA is defined as an individual's perception of a favorable outcome in adapting to cumulative physiological and functional changes associated with the passage of time. It involves experiencing spiritual connectedness and a sense of meaning and purpose in life (4). This concept combines physiological adaptation and spiritual transcendence, highlighting the understanding and subjective feelings of older adults regarding SA. It consists of four fundamental elements: Functional performance mechanism, Intrapsychic factors, Spirituality, and Gerotranscendence. In essence, SA is a product of the biopsychosocial health model, emphasizing not only material abundance and external conditions' satisfaction but also older adults' feelings about their lifestyle and physical and mental state. The orientation and perception of older adults regarding whether they have achieved their desired state of wellbeing in old age constitute Successful Aging. Promoting SA within an aging society serves a dual purpose: enhancing the quality of life for older adults and alleviating the societal burden of governance (5). Consequently, investigating the impact pathway of SA holds considerable strategic importance for fostering healthy aging in the population and addressing the challenges associated with older adults care.

Previous research indicates that social support (SS) and meaning in life (MIL) serve as crucial protective elements for SA. The primary effects model of SS suggests that a greater perception of SS corresponds to the development of more positive life intentions (6). Consequently, MIL may act as a mediator in the relationship between SS and SA. Han Jing's study delved into the mechanisms through which SS impacts SA, focusing on loneliness as a mediating variable (7). However, no study has explored MIL as a mediator and frailty as a moderator. The Selection Optimization and Compensation (SOC) model of SA posits that achieving SA involves balancing the negative energy lost in older adults with the positive energy gained, with “loss” such as frailty and physical decline and “gain” such as MIL playing protective roles (8). Aligning with the main effect model of SS and the SOC model of SA, this study concentrates on the psychosocial factors of SS and MIL. Its objective is to examine the mediating role of MIL between SS and SA, along with the moderating impact of frailty in this mediation model. The findings aim to offer targeted guidance for enhancing SA among older adults in nursing homes and promoting healthy aging.

### 1.1 SS and SA

SS encompasses assistance from individuals, groups, and communities, serving as a crucial external resource that contributes to positive experiences, stress reduction, and overall wellbeing throughout an individual's lifespan. As outlined by Xiao, SS comprises three facets: objective support, subjective support, and support utilization. Objective support involves material assistance, the existence of social networks, and participation in group relationships. Subjective support pertains to the emotional experiences and satisfaction of individuals feeling respected, supported, and understood in society. Support utilization refers to the extent to which individuals make use of SS (9).

Drawing from the regulation model of lifelong development theory and the adaptive function of control strategies, SA can be understood as an individual's capacity to access long-term self-coordination. Individuals can optimize lifelong development management by enhancing resource inputs and employing compensatory strategies to regulate lifelong development successfully (10). SS, as a vital external resource, plays a positive role in facilitating long-term access to resources or potentials, counteracting intrinsic aging processes, and thereby promoting SA. Conversely, a lack of SS resources hinders the regulation of lifelong development, negatively impacting the SA process.

Empirical studies support the significant impact of SS on SA (11). For instance, a survey involving 210 sexual minority men over 50 highlighted high levels of SS as crucial protective factors for SA. Among older sexual minority men, maintaining social contacts, reducing loneliness, and improving the quality of life were identified as determinants of SA (12). A longitudinal study of 230 adults aged 36–65 (134 with HIV, 96 without HIV) revealed that a substantial increase in SS correlated with enhanced SA. High SS was found to improve SA among individuals with HIV and older adults, even in the face of declining health status (13). These findings underscore SS as a significant predictor of SA, forming the basis for Hypothesis 1.

Hypothesis 1: There is a significant positive relationship between SS and SA.

### 1.2 The mediating role of MIL

The concept of Meaning in Life (MIL) was initially introduced by psychiatrist Prof. Frankl, denoting an individual's perception of the purpose and value of their existence. MIL encompasses a sense of life's value and fulfillment, comprising both the pursuit and experience of meaning (14). Frankl contends that the quest for meaning is an intrinsic motivator for life, helping individuals endure “any” pain positively and optimistically. Conversely, a lack of MIL may lead to feelings of boredom, emptiness, and adversely impact an individual's physical and mental health (14).

SA is not solely influenced by SS but is also directly connected to MIL. The mid-range nursing theory of SA posits that as individuals age, somatic functioning gradually changes. Those who can experience spiritual connectivity and perceive meaning and purpose in life are better equipped to adapt to these somatic changes, thereby achieving SA (4). Yu's study of 339 community-dwelling older adults in Chongqing, China, demonstrated an association between SA, positive mental health, and higher levels of MIL. Older adults with elevated mental health and MIL levels were more likely to exhibit optimistic attitudes and better adaptability to life, constituting important protective factors for promoting SA (15). Similarly, Huiping's study of 417 retired teachers in Kunming revealed a significant positive correlation between MIL and SA, suggesting that a greater sense of meaning in life increases the likelihood of achieving SA (16).

Moreover, there exists a close correlation between SS and MIL. The main effect model of SS posits that SS has a positive gain effect on individual development. Increased SS perception leads to positive life intentions, heightened positive emotions, greater enthusiasm for life, and an active pursuit of the meaning and value of life, fostering a sense of belonging, fulfillment, and significance (17). Conversely, decreased SS perception is associated with negative emotions, such as loneliness and despondency, and a reduced likelihood of experiencing positive aspects of MIL. Empirical studies affirm the impact of SS on MIL, indicating that good SS is fundamental for enhancing MIL and serves as a motivational source for exploring the meaning of life (18). Other studies suggest that SS influences older adults' MIL, with positive social relationships promoting MIL development (19, 20). Lin's study of 215 older adults revealed a positive correlation between SS and MIL (21). Martela's research further confirmed that SS independently influences MIL (22). Thus, both theoretical and empirical studies affirm the correlation between perceived SS and MIL. Higher SS levels contribute to increased MIL, potentially mediating the relationship between SS and SA. Based on this, Hypothesis 2 is proposed.

Hypothesis 2: MIL mediates the relationship between SS and SA.

### 1.3 The moderating role of frailty

MIL is a psychosocial variable that has the potential to contribute to improved SA and positively predict SA outcomes. However, it is observed that not all older adults in nursing homes with the same MIL exhibit the same level of SA. This variability could be attributed to various factors, including physiological and psychological aspects of older adults. MIL may be moderated by other factors, particularly in the case of older adults with poor health. The significance of the positive effect of MIL on SA remains a subject of debate.

Frailty is characterized as a complex clinical state involving acute changes in the health status of the organism, diminished ability to maintain homeostasis, reduced resistance to disease and stress due to age-related declines in reserve capacity, and deterioration in the functioning of multiple organ systems (23). The theory of active aging underscores that health is fundamental to active aging, and higher degrees of frailty and poorer health in older adults are less conducive to SA. The SOC model of SA emphasizes that despite experiencing resource losses during aging, individuals also gain opportunities and positive energy. The key to SA lies in balancing the negative energy lost with the positive energy gained. In the context of aging, frailty, and declining physical functioning, “gains” such as MIL and family care can play protective roles, guiding older adults toward successful aging (8).

A Swedish study with 618 older adults confirmed a significant negative correlation between frailty and MIL (24), indicating that higher levels of meaning are associated with better health. Conversely, more debilitated older adults tend to perceive themselves as more useless, resulting in lower MIL. Gordon's research suggests that frailty is a crucial factor influencing SA, and interventions to alleviate frailty in older adults can contribute to SA (25). Additionally, Huiping explored the path of MIL on SA using psychological capital as a mediator and age as a moderator among retired teachers (16), yet no study has investigated the correlation between MIL and frailty as a moderator variable. The moderating effect of frailty on MIL and SA warrants further investigation. In summary, a high level of frailty may weaken the protective effect of MIL on SA, while a low level of frailty may enhance the impact of MIL on SA. Frailty is proposed to play a negatively moderating role between MIL and SA among older adults in nursing homes, forming the basis for Hypothesis 3.

Hypothesis 3: Frailty negatively moderates between MIL and SA.

### 1.4 The current study

**Figure 1:**
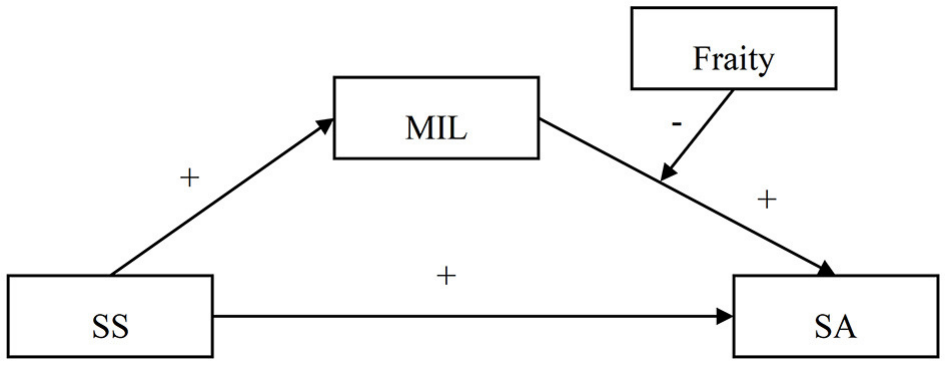
The proposed mediated moderation model.

Presently, there is a lack of comprehensive studies examining the interplay between SS, MIL, frailty, and SA. This study formulates research hypotheses based on the main effects model of SS and the SOC model of SA, with the objective of exploring a complex moderated mediation model elucidating the relationship between SS and SA in older adults residing in nursing homes (see Figure 1). The study aims to achieve the following objectives: (a) ascertain whether SS significantly predicts SA; (b) assess whether MIL mediates the relationship between SS and SA; and (c) examine whether frailty moderates the relationship between MIL and SA.

## 2 Method

### 2.1 Participants

The convenience sampling method was employed to select older adults residing in six nursing homes in Sichuan Province from March 2023 to August 2023 as participants for the study. Inclusion criteria encompassed individuals who were (1) aged 60 years or older, (2) residing in the nursing homes for at least 12 months, and (3) providing informed consent and willingly participating in the survey. Exclusion criteria involved the presence of cognitive impairment, consciousness disorder, language communication disorder, or any other conditions hindering cooperation.

A total of 600 questionnaires were distributed, and 541 were collected after excluding invalid submissions, resulting in a validity rate of 90.2%. The participant demographics were as follows: 212 males (39.2%) and 329 females (60.8%); the average age was 75.07 ± 67.03 years, with an age range of 60–92 years. Regarding education level, there were 98 individuals classified as illiterate (18.1%), 246 as elementary school graduates (45.5%), 96 as junior high school graduates (17.7%), 64 as senior high school graduates (11.8%), and 37 with specialization or higher education (6.8%). Marital status distribution consisted of 372 married individuals (68.8%), 153 widowed individuals (28.3%), and 16 divorced individuals (3.0%). In terms of the number of children, 10 individuals (1.8%) had none, 118 (21.8%) had one, 185 (34.2%) had two, and 228 (42.1%) had three or more. Regarding per capita monthly household income, 131 individuals (24.2%) had < 2,000 RMB, 234 (43.3%) had 2,000–5,000 RMB, and 176 (32.5%) had more than 5,000 RMB. The distribution of chronic diseases included 7 individuals (1.3%) with none, 109 (20.1%) with one, 201 (37.2%) with two, and 224 (41.4%) with three or more chronic diseases.

### 2.2 Procedure

This study received approval from the Ethics Committee of Deyang People's Hospital. Prior to the survey, the research objectives, significance, and instructions for completing the questionnaire were thoroughly explained to the participants. Informed consent was obtained before distributing the anonymous questionnaire, and participants were assured that their responses would be kept confidential. Throughout the survey, three investigators, all trained uniformly, conducted one-on-one surveys with older adults in nursing homes who met the inclusion and exclusion criteria.

For participants facing challenges in filling out the questionnaires due to low literacy levels or advanced age, the investigators addressed these concerns on the spot. The investigators assisted by going through the questionnaire item by item, ensuring truthful responses based on the answers provided by the patients. This approach aimed to accommodate participants with difficulties in completing the questionnaire, promoting accurate and comprehensive data collection.

## 3 Measures

### 3.1 SS

The Social Support Rate Scale (SSRS), developed by Xiao Shuiyuan, was utilized to assess the level of SS in individuals (9). The scale comprises 10 items that measure three dimensions: objective support, subjective support, and SS utilization. Items 1–4 and 8–10 are single-choice questions, with response choices assigned scores ranging from 1 to 4. Item 5 is divided into A, B, C, and D, offering a total of 4 choices, each assigned scores from 1 to 4, representing “none” to “full support”, respectively. For items 6 and 7, a score of 0 is assigned if the response is “no source”, while if the response is “the following sources”, points are awarded based on the number of sources. The total score on the SSRS is the sum of the scores from all 10 items, ranging from 12 to 66. A higher total score indicates a higher level of perceived SS. In the present study, the internal consistency of the scale was assessed using Cronbach's α coefficient, which was found to be 0.86, indicating good reliability.

### 3.2 MIL

The Meaning in Life Questionnaire (MLQ), developed by Steger et al., was employed to evaluate an individual's MIL (26). The Chinese version of MLQ, revised by Liu et al. (27), comprises 9 questions organized into two dimensions: meaning experience and meaning seeking. Respondents rate their responses on a Likert 7-point scale, ranging from 1 (no meaning at all) to 7 (very much meaning), where higher scores indicate a higher level of meaning in an individual's life. In this study, the internal consistency of the revised MLQ was assessed using Cronbach's α coefficient, which yielded a value of 0.82. This indicates good reliability for the scale in measuring MIL among the study participants.

### 3.3 SA

The assessment of SA in older adults was conducted using the Successful Aging Inventory (SAI), developed by Troutman (28). The scale comprises 20 entries distributed across 5 dimensions: inner context and meaning of existence, functional coping, transcending aging, sense of inheritance, and spirituality. Respondents provide ratings on a 5-point Likert scale, ranging from “never” to “always”. Total scores on the SAI range from 0 to 80, where a higher score reflects a higher level of Successful Aging. The Chinese version of the SAI, adapted by Cheng (29), was employed in this study, and its reliability was assessed using Cronbach's α coefficient, yielding a value of 0.81. This indicates good internal consistency for the SAI in evaluating SA among the study participants.

### 3.4 Frailty

The Tilburg Frailty Indicator (TFI), developed by Gobbens et al. at the University of Tilburg, the Netherlands, in 2010, was utilized for the self-assessment of frailty in older adults (30). The scale comprises 15 entries organized into three dimensions: physical debility, psychological debility, and social debility. Participants use a dichotomous scoring method (0–1 points) for each entry, resulting in a total score ranging from 0 to 15. A score of ≥5 points is considered indicative of frailty, with higher scores indicating a more severe degree of debilitation. The Cronbach's alpha coefficient for the TFI is reported as 0.74. The Chinese version of the TFI, adapted by Xi Xing in 2013, was utilized in this study, with its Cronbach's alpha coefficient reported as 0.686 (31). In the present study, the internal consistency of the TFI was assessed using Cronbach's α coefficient, yielding a value of 0.64. While slightly lower than the reported values, this still suggests an acceptable level of reliability for the TFI in evaluating frailty among the study participants.

### 3.5 Data analysis

Statistical analyses utilized SPSS 26.0. Common method bias was assessed employing Harman's single-factor test within the exploratory factor analysis approach. P-P plots and Q-Q plots were employed to ascertain the departure of data from normal distribution. Descriptive analyses featured median (P25, P75), while Spearman correlation analyses examined the relationships among SS, MIL, frailty, and aging correlation. For mediation analysis, Hayes' PROCESS 4.1 macro program's Model 4 assessed the mediating effect of MIL on the SS to SA relationship. Model 14 examined frailty's moderating effect on the second half path of the mediation model (32). A simple slope approach was employed to analyze frailty's moderating role between MIL and SA. Significant differences in total SA scores among older adults in nursing homes were observed for age and marital status (*Z* = 8.935, *p* < 0.05; *Z* = 6.299, *p* < 0.05). Consequently, gender and age served as control variables. All tests utilized the bias-corrected percentile Bootstrap method, repeated 5,000 times, calculating 95% confidence intervals. Significance was determined if confidence intervals for the mediation effect, moderated mediation effect, and moderated effect did not include 0 (33). The test level was set at α = 0.05.

## 4 Results

### 4.1 Common method biases test

The exploratory factor analysis method in Harman's single-factor test was employed to validate the reliability and accuracy of the data. Findings revealed 16 factors with eigenroot values exceeding 1 in the unrotated condition. The 1st factor explained 15.78% of the variance, falling below the critical 40% criterion. This outcome indicates the absence of significant common method bias in the study's data (34).

### 4.2 Preliminary analysis

Table 1 displays the median, quartiles, and correlations among study variables. SS in older adults from nursing homes exhibited positive correlations with MIL (*r* = 0.371, *P* < 0.01) and SA (*r* = 0.411, *P* < 0.01), and a negative correlation with frailty (*r* = −0.380, *P* < 0.01). MIL demonstrated positive correlations with SA (*r* = 0.538, *P* < 0.01) and negative correlations with frailty (*r* = −0.399, *P* < 0.01). Additionally, SA was negatively correlated with frailty (*r* = −0.382, *P* < 0.01).

**Table 1:** Descriptive statistics and correlations for all variables.

**Variables**	**SS**	**MIL**	**SA**	**Frailty**
SS	1			
MIL	0.371^**^	1		
SA	0.411^**^	0.482^**^	1	
Frailty	−0.380^**^	−0.399^**^	−0.382^**^	1
*M* (P25, P75)	37.00 (30.00, 43.50)	35.00 (29.00, 42.00)	50.00 (42.00, 58.50)	5.00 (3.00, 7.00)

^**^p < 0.01.

SS, Social Support; MIL, Meaning In Life; SA, Successful Aging.

### 4.3 Mediation effect of MIL

To assess Hypothesis 2, Model 4 in the PROCESS 4.1 macro program was employed to examine the mediating role of MIL between SS and SA. As indicated in Table 2, with age and marital status controlled for, SS in older adults from nursing homes positively predicted SA (β = 0.400, *P* < 0.001) and positively predicted MIL (β = 0.339, *P* < 0.001). Additionally, MIL positively predicted SA in older adults from nursing homes (β = 0.374, *P* < 0.001). The direct effect of SS on SA was significant (β = 0.273, *p* < 0.001).

**Table 2:** Testing the mediating effect of MIL between SS and SA.

	**Model 1**		**Model 2**		**Model 3**	
	**SA**		**MIL**		**SA**	
	β	***t***	β	***t***	β	***t***
Age	−0.092	−1.595	−0.132	−2.231^*^	−0.043	−0.801
Marital status	0.071	0.945	−0.081	−1.055	0.101	1.454
SS	0.400	10.046^***^	0.339	8.347^***^	0.273	6.979^***^
MIL	——	——	——	——	0.374	9.545^***^
*R^2^*	0.172		0.139		0.292	
*F*	37.152		28.792		55.313	

^*^p < 0.05.

^***^p < 0.001.

SS, Social Support; MIL, Meaning In Life; SA, Successful Aging.

Using the bias-corrected Bootstrap method with 5,000 repetitive samples, the results revealed a significant indirect effect of SS on SA among older adults in nursing homes through MIL: *b* = 0.127, *SE* = 0.020, 95%CI = (0.089, 0.168). The direct effect of SS (0.273) and the mediating effect of MIL (0.127) accounted for 68.25 and 31.75% of the total effect, respectively. Therefore, the mediating effect of MIL on the relationship between SS and SA supports Hypothesis 2.

### 4.4 Moderated mediation

**Figure 2:**
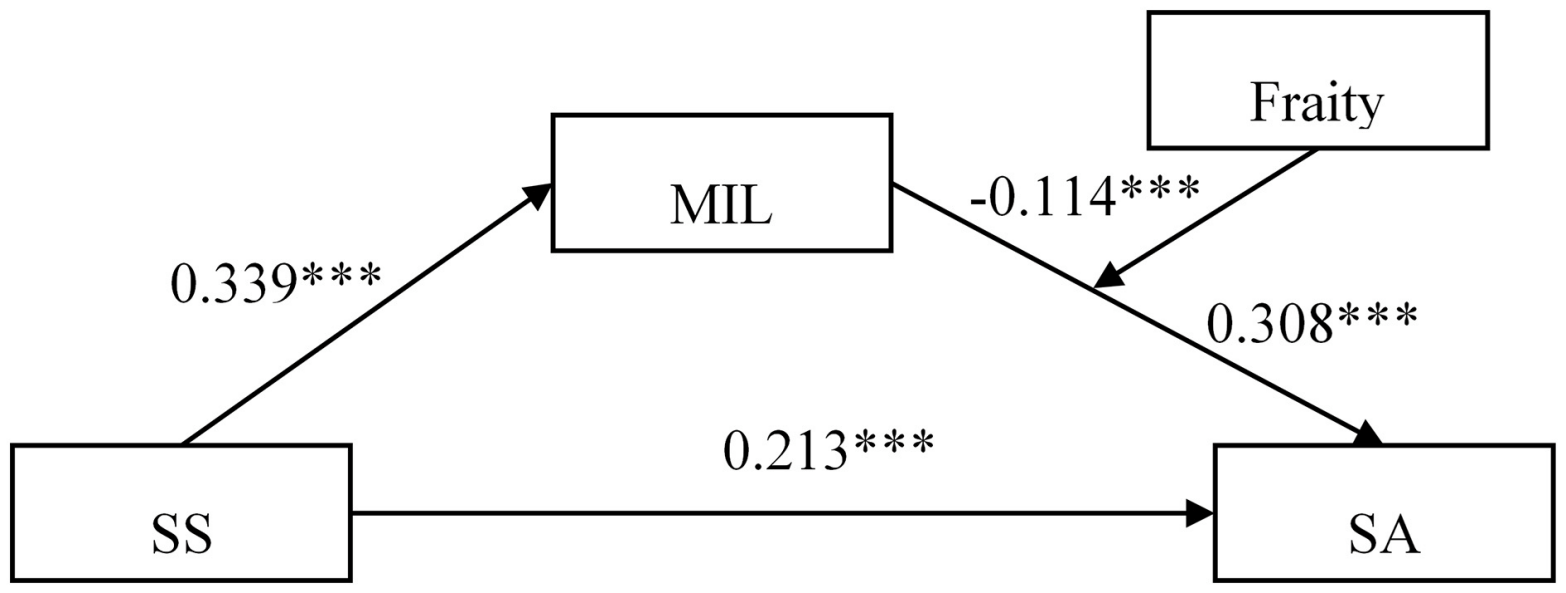
Mediated moderation model. ***p < 0.001.

To examine Hypothesis 3, Model 14 within the PROCESS 4.1 macro program was utilized to assess the moderated mediation effect of frailty on MIL and SA. As presented in Table 3 and Figure 2, with age and marital status controlled for, MIL and frailty exhibited a significant interaction effect on SA among older adults in nursing homes (β = −0.114, *p* = 0.008). A bias-corrected Bootstrap method with 5,000 repetitions indicated that the 95% confidence intervals for MIL and the moderating cross-sectional items did not include 0 [95%CI = (−0.197, −0.030)]. Frailty demonstrated a moderating effect on the indirect effects of SS and SA in older adults in nursing homes, with a moderating index of −0.039, *SE* = 0.020, and 95%CI = (−0.082, −0.003). MIL significantly mediated the relationship between SS and SA in older adults in nursing homes when frailty was low (one standard deviation below the mean): *ab* = 0.143, 95%CI = (0.097, 0.196). Simultaneously, MIL significantly mediated the relationship between SS and SA in older adults in nursing homes when frailty was high (one standard deviation above the mean): *ab* = 0.066, 95%CI = (0.010, 0.125).

**Table 3:** Testing moderated mediation effect of acceptance on SS and SA.

	**Model 1**		**Model 2**	
	**MIL**		**SA**	
	**β**	***t***	**β**	***t***
Age	−0.132	−2.231^*^	0.002	0.030
Marital status	−0.081	−1.055	0.142	2.062^*^
SS	0.339	8.347^***^	0.213	5.241^***^
MIL	——	——	0.308	7.495^***^
Frailty	——	——	−0.192	−4.455^***^
MIL × Frailty	——	——	−0.114	−2.663^**^
*R^2^*	0.139		0.321	
*F*	28.792		42.07	

^*^p < 0.05.

^**^p < 0.01.

^***^p < 0.001.

SS, Social Support; MIL, Meaning In Life; SA, Successful Aging.

**Figure 3:**
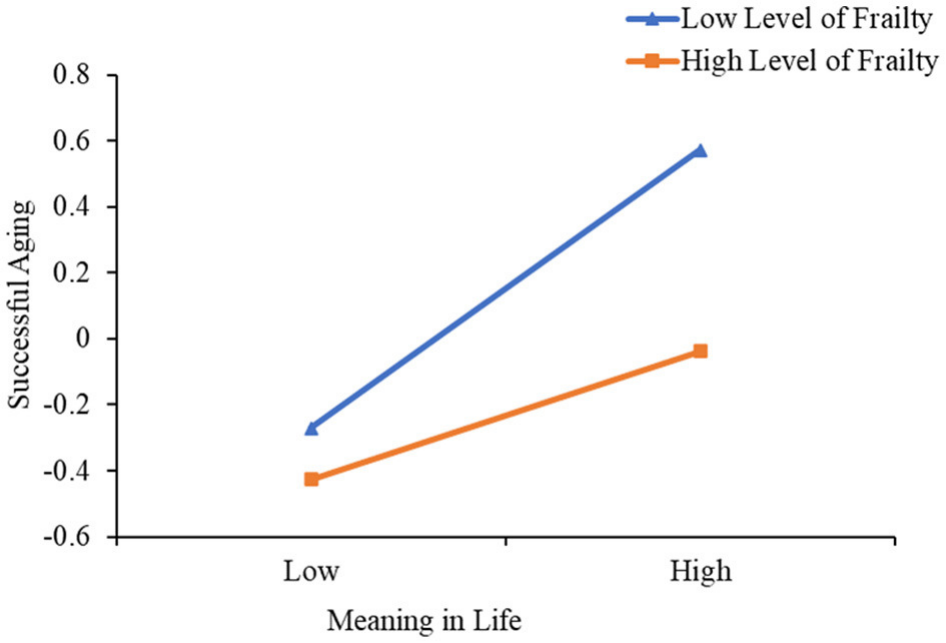
Model of test for simple slopes showing moderating influence of frailty in the association between MIL and SA.

To enhance comprehension of the moderating impact of frailty on MIL and SA, analyses were performed using a simple slope test. As depicted in Figure 3, when frailty was at a low level (*M* – 1SD), MIL of older adults in nursing homes significantly positively predicted SA (βsimple = 0.422, *P* < 0.001). The positive predictive effect of MIL on SA was also significant (βsimple = 0.194, *p* = 0.004) when the level of frailty was high (*M* + 1SD), though the predictive effect was relatively diminished. Consequently, the predictive influence of SS on MIL tended to decrease as frailty increased, indicating that frailty played a moderating role in the effect of MIL on SA.

## 5 Discussion

Aging represents an inexorable aspect of population development. As the extent of population aging intensifies, the promotion of SA and the alleviation of the burden of social old-age care have emerged as focal points of societal concern. While several empirical studies have delved into the mechanisms through which SS influences SA, a limited number of investigations have considered the mediating variable of a sense of MIL in the impact of SS on SA among older adults residing in nursing homes. Drawing on the main effects model of SS and the SOC model of SA, this current study underscores the significance of two psychosocial factors—SS and MIL. It specifically explores the mediating role of MIL and the moderating influence of frailty in the relationship between SS and SA among older adults in nursing homes.

### 5.1 SS and SA

The findings of the present study revealed a significant negative correlation between SS and SA among older adults in nursing homes, corroborating Hypothesis 1 and aligning with previous research results. Past investigations have consistently demonstrated that robust social support significantly contributes to enhancing SA in older adults (12, 13). On one hand, older adults in nursing homes with higher levels of SS tend to seek external assistance when facing challenges, receiving diverse support sources. This abundance of resources equips them to effectively navigate daily life challenges, thereby reducing psychological stress, alleviating mental strain, mitigating negative emotions, enhancing social adaptability, and maintaining physical and mental wellbeing (7). Such positive adaptations facilitate better adjustment to the life of older adults, thereby promoting the attainment of successful aging. On the other hand, in accordance with the regulation model of Lifespan Development Theory and the adaptive function of control strategies, individuals necessitate adaptive self-regulation and optimization of Lifespan Development management to achieve successful aging by enhancing resource input and applying the varied effects of different control strategies (10). Conversely, individuals with lower levels of SS receive fewer support resources, impeding the process of successful aging and diminishing the regulation of lifelong development. This, in turn, adversely affects the level of successful aging among older adults in nursing homes. In essence, the lower the level of SS, the poorer the regulation of lifelong development, hindering the process of successful aging. Especially after the pandemic, 35.86% of the general population sometimes or often feel lonely (35). For older adults in nursing homes, the inability of their children to accompany them, reduced exposure to social interactions, and decreased social support contribute to heightened feelings of loneliness. This exacerbates the accumulation of loneliness, and older adults experiencing severe loneliness may not proactively seek help and support when facing problems and difficulties. This forms a vicious circle that further diminishes their level of SA.

### 5.2 Mediation effect of MIL

The current study's outcomes lend support to Hypothesis 2, affirming that a sense of MIL acts as a mediating factor in the connection between SS and SA among older adults in nursing homes. This finding is consistent with prior research that identifies SS as a significant predictor of MIL (19, 20, 36, 37) and underscores MIL as a vital predictor of SA (15, 16).

In line with the main effects model of SS, which posits a generalized positive impact on emotional experience, physical and psychological wellbeing, irrespective of the current level of support, SS proves advantageous to an individual's psychological wellbeing (6). Concurrently, the maintenance model of MIL asserts that the structure and function of social relationships influence an individual's MIL. Understanding social support as a social resource aids individuals in realizing MIL, fostering goal achievement, a sense of belonging, identity, and overall enhancement of MIL (38). The perception of greater SS by older adults in nursing homes equips them to effectively confront environmental challenges, actively pursue and realize the significance of life, and promote a meaningful understanding of life, ultimately enhancing their MIL. Conversely, reduced SS perception leads to heightened negative experiences of loneliness, loss, and depression, limiting opportunities for MIL realization and diminishing positive MIL experiences.

Research underscores MIL as a pivotal source of an individual's mental health, psychological wellbeing, and sense of personal worth and existence (39). It plays a crucial role in stress regulation, mental health promotion, and positive coping with illness (40). According to Frankl's theory of MIL and Flood's mid-range nursing theory of SA (4, 14), MIL stands as the most critical component of an individual's spiritual dimension. Higher MIL levels in older adults in nursing homes facilitate the mobilization of positive emotions, alleviation of negative emotions like loneliness, anxiety, and depression, and the maintenance of adaptive psychology and behaviors, ultimately contributing to the achievement of successful aging. Conversely, lower MIL levels result in increased discomfort and helplessness in dealing with stressful events, negatively impacting the physical and mental health of individuals and hindering the pursuit of successful aging.

In summary, the study establishes that SS predicts MIL and SA, MIL predicts SA, and MIL serves as a mediating factor in the relationship between SS and SA among older adults in nursing homes.

### 5.3 The moderating role of frailty

The findings of the current study supported Hypothesis 3, revealing that frailty among institutionalized older adults moderated the latter part of the indirect effect of SS on SA, specifically moderating the relationship between a sense of MIL and SA. The correlation between MIL and SA was more pronounced among older adults in nursing homes with lower levels of frailty. Conversely, this correlation weakened among those with higher levels of frailty. Essentially, as the debilitation of older adults in nursing homes increased, the influence of MIL on SA diminished. The impact of MIL on SA was stronger in individuals with lower frailty levels, indicating that more debilitated older adults experienced a reduced effect of MIL on SA.

Moreover, in accordance with the theory of lifelong development and the SOC model of SA, aging leads to the loss of various resources, both physical and psychological, resulting in an escalating degree of frailty. Nonetheless, older adults can attain successful aging by effectively regulating lifelong development, strategically deploying and optimizing acquired resources such as MIL (8, 10). As frailty deepens, the frequency and amplitude of resource allocation widen in the process of resource selection, compensation, and optimization. This serves to compensate for the losses incurred due to frailty, making it more challenging for individuals to achieve successful aging through the acquisition of resources like MIL.

In summary, higher levels of frailty among older adults in nursing homes diminish the impact of MIL on successful aging.

## 6 Limitations

Building upon the main effects model of SS and the SOC model of SA, the present study delves into a moderated mediation effects model concerning older adults in nursing homes. The study discerns that a sense of MIL serves as a partial mediator in the relationship between SS and SA. Additionally, frailty emerges as a factor capable of diminishing the positive predictive impact of MIL on SA. Frailty also moderates the latter part of the pathway in the mediation model, wherein SS influences SA through MIL. These findings offer theoretical rationale for the development of interventions aimed at enhancing SA in institutionalized older adults. However, it is essential to acknowledge certain limitations in this study. Firstly, owing to constraints in human, material, and financial resources, a cross-sectional survey was employed. Consequently, the findings only provide a snapshot of the situation at that specific point in time, and it is not possible to establish the order of temporal changes among the variables, therefore, no inference can be made about causality, Nevertheless, the impact of SS on SA in older adults residing in nursing homes may constitute a dynamic and prolonged process. Future research could benefit from adopting a longitudinal research approach to capture the evolving nature of this relationship over time. Secondly, it's important to acknowledge that the pathway of the influence of SS on SA may encompass the effects of other physical, psychological, and social factors. Factors such as physical activity, self-efficacy, and subjective economic status could play significant roles in shaping the overall impact of SS on the successful aging process. Further exploration of these potential contributing factors could enhance our understanding of the complex interplay involved in promoting successful aging among institutionalized older adults. Thirdly, this study relied on self-reported questionnaires from older adults, lacking measures of objective indicators such as cognitive skills, physical activity limitations, and physical fitness. Consequently, there may be some bias in the findings; Fourth, it's important to note that the sample in the current study comprises only older adults from six nursing homes in a single province in China. Consequently, the representativeness of the sample is somewhat restricted. Future research endeavors should consider broadening the survey area and expanding the sample size to enhance the overall representativeness of the study population. This approach will contribute to a more comprehensive understanding of the factors influencing successful aging among older adults in diverse regions and settings.

## 7 Implications for practice

Despite these limitations, the current study offers valuable insights for developing intervention strategies aimed at enhancing SA among older adults in nursing homes. First and foremost, the study underscores the role of a sense of MIL as a mediating variable between SS and SA. Improving the MIL of older adults in nursing homes holds the potential to elevate their levels of SA. In the context of long-term care, nursing home staff should not only provide essential medical assistance but also recognize the significance of “spiritual aging”. This involves prioritizing the spiritual wellbeing of older adults, encouraging their participation in social and cultural activities, and providing additional support and care to help them experience MIL more frequently. Secondly, it is crucial to recognize that frailty is a reversible condition. Mitigating frailty among older adults in nursing homes can amplify the positive impact of MIL on SA. To achieve this, nursing home personnel should focus on screening for frailty indicators among older adults, bolster psychological support, and implement physical function exercises for those with higher frailty levels. This proactive approach can contribute to delaying the onset of frailty among older adults, thereby promoting successful aging in nursing home settings.

## 8 Conclusion

This study, grounded in the main effects model of SS and the SOC model of SA, focused on examining the influence of two psychosocial factors, SS and a sense of MIL, on SA. Additionally, it explored the moderating role of frailty between MIL and SA, elucidating the interplay of MIL and frailty in the relationship between SS and SA among older adults in nursing homes. The results underscore that MIL serves as a mediating variable between SS and SA. Furthermore, frailty emerges as a moderator between MIL and SA, potentially diminishing the positive impact of MIL on SA. This study provides valuable insights for directing efforts aimed at promoting successful aging among older adults in nursing homes.

## Data availability statement

The raw data supporting the conclusions of this article will be made available by the authors, without undue reservation.

## Ethics statement

The studies involving humans were approved by Ethics Committee of Deyang People's Hospital. The studies were conducted in accordance with the local legislation and institutional requirements. The participants provided their written informed consent to participate in this study. Written informed consent was obtained from the individual(s) for the publication of any potentially identifiable images or data included in this article.

## Author contributions

FX: Data curation, Investigation, Writing – original draft, Writing – review & editing. JZ: Data curation, Funding acquisition, Investigation, Writing – review & editing. SX: Data curation, Investigation, Writing – review & editing. QL: Investigation, Writing – review & editing. YL: Investigation, Writing – review & editing.
