# Supplementary material for: The impact of social support for older adults in nursing homes on successful aging: a moderated mediation model
Source: Front Public Health. 2024 Feb 21;12:1351953. doi: 10.3389/fpubh.2024.1351953 (PMC10916522; doi:10.3389/fpubh.2024.1351953)
Supplement: Supplementary file 1 [file Table_1.docx]

**Scale Scoring Method （****English Version）**

This is a description of the article "The impact of social support for older adults in nursing homes on successful aging: a moderated mediation model" (Manuscript ID: 1351953). The scoring methodology of the scales used in this article will help us to accurately calculate the total and dimensional scores of the scales after they have been used in a survey.

# Social Support: Social Support Rate Scale, SSRS

It consists of 10 questions measuring three dimensions: objective support, subjective support, and social support utilization. Topics 1 to 4 and 8 to 10 were single choice, with 1 to 4 points for choosing 1 to 4 items; topic 5 was divided into A, B, C, and D, with a total of 4 choices, with 1 to 4 points for each item from "none" to "full support"; topics 6 and 7 scored 0 points for "no source" and 0 points for "the following sources", with several sources for those who answered "none of the following sources"; and topics 6 and 7 scored 0 points for "no source" and 0 points for "the following sources". For questions 6 and 7, if you answered "no source", it was 0 points, and if you answered "the following sources", you were given several points for each source.

Total Score: sum of 10 questions, total score range 12-66; Objective Support Score: sum of 2, 6, and 7 question ratings; Subjective Support Score: sum of 1, 3, 4, and 5 question ratings; Social Support Utilization: sum of 8, 9, and 10 question ratings^[1]^.

# Meaning in Life: Meaning in Life Questionnaire, MLQ

A total of 9 questions, categorized into two dimensions, meaning experience and meaning seeking, on a 7-point Likert scale ranging from 1 (no meaning at all) to 7 (very much meaning), with higher scores indicating higher levels of individuals' sense of meaning in life

Total: sum of 9 questions, total score range 9 to 63; Meaningful Experience: sum of questions 1 to 5; Meaning Seeking: sum of questions 6 to 9^[2]^.

# Successful Aging: Successful Aging Inventory, SAI

A total of 20 questions, including 5 dimensions of inner context and meaning of existence (8 entries), functional coping (5 entries), transcending aging (4 entries), sense of inheritance (1 entry), and spirituality (2 entries), were scored on a 5-point Likert scale ranging from 0 to 4 on a scale of "never" to " always." Higher scores indicate higher levels of successful aging. The Likert 5-point scale was used, from "never" to "always", with scores ranging from 0 to 4. The higher the score, the higher the level of successful aging of the elderly.

Total Score: Sum of 20 questions, total score range 0-80; Inner Context and Meaning of Being: sum of questions 3, 7, 8, 9, 17, 18, 19, 10; Functional Coping: sum of questions 1, 2, 4, 5, 6;

Transcending Aging: sum of questions 10, 12, 13, 14, Sense of Legacy: score for question 16; Spirituality: sum of questions 11, 15^[3]^.

1. **Frailty: Tilburg Frailty Indicator (TFI)**

A total of 15 questions, including 3 dimensions of somatic debility, psychological debility, and social debility, were scored using a dichotomous scoring system (0 to 1), with higher scores indicating a more severe degree of debility in the individual.

Total score: sum of 15 questions, total score range 0-15, ≥5 judged debilitating; somatic debilitation: sum of questions 1-8; psychological debilitation: sum of questions 9-12; social debilitation: sum of questions 13-15^[4]^.

References

[1] Xiao SY. Theoretical basis and research application of ‘social support rating scale’. J *Clin Psychiatry*. (1994) 2:98–100.

[2] Liu SS, Gan YQ. Reliability and validity of Chinese version of meaningin life scale among college students. *Chin J Mental Health*. (2010) 24:478–82. doi: 10.3969/j.issn.1000-6729.2010.06.021

[3] Troutman M, Nies MA, Small S, Bates A. The development and testing of an instrument to measure successful aging. *Res Gerontol Nurs*. (2011) 4:221–32. doi: 10.3928/19404921-20110106-02

[4] Xi X, Guo GF, Sun J. Study on the reliability and validity of the Chinese version of tilburg frailty assessment scale. *J Nurs*. (2013)20:1–5. doi: 10.16460/j.issn1008-9969.2013.16.006
