# Supplementary material for: The impact of social support for older adults in nursing homes on successful aging: a moderated mediation model
Source: Front Public Health. 2024 Feb 21;12:1351953. doi: 10.3389/fpubh.2024.1351953 (PMC10916522; doi:10.3389/fpubh.2024.1351953)
Supplement: Supplementary file 2 [file Table_2.docx]

**Scale Scoring Method****（****Chinese Version）**

This is a description of the article "The impact of social support for older adults in nursing homes on successful aging: a moderated mediation model" (Manuscript ID: 1351953). The scoring methodology of the scales used in this article will help us to accurately calculate the total and dimensional scores of the scales after they have been used in a survey.

**1. Social Support：Social Support Rate Scale，SSRS**

由10个题目组成，衡量三个维度：客观支持、主观支持、社会支持利用度。第1~4、第8~10 题目为单选，选择1~4 项分别计1~4 分；第5 题目分A、B、C、D 共4 个选项，每项从“无”到“全力支持”分别计1~4 分；第6、第7 题目如回答“无任何来源”计0 分，回答“下列来源”者，有几个来源计几分。

总分：10 个题目计分之和，总分范围为12~66分；客观支持得分:2，6，7题评分之和;主观支持得分：1，3，4，5题评分之和；社会支持利用度：8，9，10题评分之和^[1]^。

**2.Meaning in Life：Meaning in Life Questionnaire，MLQ**

共9个题目，分为意义体验和意义寻求两个维度，采用Likert 7点计分法，范围从 1（完全没有意义）到 7（非常多意义），得分越高表明个体的生命意义感水平越高

总分：9个题目之和，总分范围9~63分；意义体验：1~5题之和；意义寻求：6~9题之和^[2]^。

**3. Successful Aging： Successful Aging Inventory，SAI**

共20 个题目，包括内心情境和生存意义（8 个条目）、功能性应对（5 个条目）、超越老化（4 个条目）、传承感（1 个条目）和精神性（2 个条目）5 个维度，采用Likert 5级评分法，从“从不”到“总是”依次计为0～4 分，得分越高表示老年人的成功老龄化的水平越高。

总分：20 个题目之和，总分范围0～80 分；内心情境和生存意义：3，7，8，9，17，18，19，10题之和；功能性应对：1，2，4，5，6题之和；超越老化：10，12，13，14题之和，传承感：第16题的得分；精神性：11，15题之和^[3]^。

**4.Frailty ：Tilburg Frailty Indicator，TFI）**

共15个题目，包括躯体衰弱、心理衰弱、社会衰弱3个维度，采用二分类计分法（0~1分），分数越高表示个体的衰弱程度越严重。

总分：15个题目之和，总分范围0~15分，≥5分判定为衰弱；躯体衰弱：1~8题之和；心理衰弱：9~12题之和；社会衰弱：13~15题之和^[4]^。

References

[1] Xiao SY. Theoretical basis and research application of ‘social support rating scale’. J *Clin Psychiatry*. (1994) 2:98–100.

[2] Liu SS, Gan YQ. Reliability and validity of Chinese version of meaningin life scale among college students. *Chin J Mental Health*. (2010) 24:478–82. doi: 10.3969/j.issn.1000-6729.2010.06.021

[3] Troutman M, Nies MA, Small S, Bates A. The development and testing of an instrument to measure successful aging. *Res Gerontol Nurs*. (2011) 4:221–32. doi: 10.3928/19404921-20110106-02

[4] Xi X, Guo GF, Sun J. Study on the reliability and validity of the Chinese version of tilburg frailty assessment scale. *J Nurs*. (2013)20:1–5. doi: 10.16460/j.issn1008-9969.2013.16.006
