# Supplementary material for: The impact of social support for older adults in nursing homes on successful aging: a moderated mediation model
Source: Front Public Health. 2024 Feb 21;12:1351953. doi: 10.3389/fpubh.2024.1351953 (PMC10916522; doi:10.3389/fpubh.2024.1351953)
Supplement: Supplementary file 3 [file Table_3.docx]

**The Chinese version of the questionnaires**

This is a paper version of the scale used in the article "The impact of social support for older adults in nursing homes on successful aging: a moderated mediation model" (Manuscript ID: 1351953).We used this questionnaire in our survey of older adults in nursing homes.

**养老机构老年人成功老龄化问卷及相关因素调查表**

您好，感谢您百忙之中抽空填写此份问卷。我们是德阳市人民医院的研究者，这是一份关于老年人成功老龄化的问卷，本次调查以无记名方式进行，您回答的信息我们会高度保密，不会透露给他人，谢谢您的配合与支持！

**一、一般资料调查表**

**指导语：以下问题旨在调查您的一般资料，请在每小题后相应的方框内打“√”。**

1.您的性别 ：□男 □女

2.您的年龄 岁

3.教育水平 ：

□文盲 □小学 □初中 □高中 □专科及以上

4.婚姻状况 ：

□已婚 □离异 □丧偶 □未婚

5.子女数量：

□无 □1个 □2个 □3个及以上

6.家庭人均月收入 ：

□＜2000元 □2000-5000元 □＞5000元

7.生活方式

7.1饮酒：

□从不饮酒 □偶尔饮酒 □经常饮酒

7.2吸烟 ：

□有吸烟习惯 □无吸烟习惯或戒烟

7.3饮

□素食为主 □肉食为主 □荤素合理搭配

7.4锻炼 ：

□很少 □有时 □经常

7.5睡眠：

□正常 □有时失眠 □经常失眠

8.慢性疾病：请标出你已被医院确诊的所有慢性疾病（多选题）。

□高血压 □心脏病 □糖尿病 □脑血管疾病

□关节炎 □癌症 □颈椎病 □哮喘

□慢性支气管炎 □肺气肿 □消化性溃疡病 □白内障

□肝硬化 □肾衰竭 □其他慢性疾病 ____________（如有，请补充）

**二、衰弱指数**

**指导语：以下问题旨在了解您现在的衰弱情况，请在每小题相应的方框内打“√”。**

**第一部分：身体功能方面**

1. 您觉得自己身体健康吗 ？

□是 □否

2. 您的体重是否下降了很多（最近6个月下降达6kg以上，或最近一个月下降达3kg以上，排除可以减轻体重 ）？

□是 □否

3. 是否由于行走困难影响您的日常生活

□是 □否

4.是否由于保持平衡困难影响您的日常生活

□是 □否

5.是否由于听力差影响您的日常生活？

□是 □否

6.是否由于视力不好影响您的日常生活？

□是 □否

7. 是否由于手没劲影响您的日常生活？

□是 □否

8. 是否由于身体疲乏影响您的日常生活？

□是 □否

**第二部分：心理方面**

9. 您记忆力有没有问题？

□是 □有时 □否

10. 您最近一个月有没有感到情绪低落？

□是 □有时 □否

11. 您最近一个月有没有感到紧张或焦虑？

□是 □有时 □否

12. 您能很好地处理遇到的问题吗？

□是 □有时 □否

**第三部分：社会方面**

13. 您是否独居？

□是 □否

14. 希望有人陪伴在您身边？

□是 □否

15. 您是否可以从他人那里得到足够的帮助？

□是 □否

**三、成功老龄化问卷**

**指导语：以下问题旨在了解您现在的成功老龄化情况，请在每小题后的“从不、偶尔、有时、经常、总是”相应的表格内打“√”。**

| 项目 | 从不 | 偶尔 | 有时 | 经常 | 总是 |
| --- | --- | --- | --- | --- | --- |
| 1.我能处理家里需要我处理的事情以及照顾好自己(吃饭、洗澡、穿衣等) |  |  |  |  |  |
| 2.到目前为止，我能应对随着年龄增长而带来的身体上的变化 |  |  |  |  |  |
| 3.我对未来的生活很乐观 |  |  |  |  |  |
| 4.我感觉我能应对年龄变老的问题 |  |  |  |  |  |
| 5.我感觉我能处理生活中的问题 |  |  |  |  |  |
| 6.我能想到问题的解决方法 |  |  |  |  |  |
| 7.我善于思考解决问题的新方法 |  |  |  |  |  |
| 8.我喜欢尝试新鲜事物 |  |  |  |  |  |
| 9.我乐观开朗 |  |  |  |  |  |
| 10.我思念已经过世的亲人并感觉和他们距离很近 |  |  |  |  |  |
| 11.我花一些时间祷告或参加一些宗教活动 |  |  |  |  |  |
| 12.随着年龄的增长，我思考世界的方式改变了 |  |  |  |  |  |
| 13.我希望有几个亲密的朋友而不是很多普通朋友 |  |  |  |  |  |
| 14.对于一个问题或情况，有时候会有两个正确的答案 |  |  |  |  |  |
| 15.有一种信仰对于我来说非常重要(例如共产主义、佛教、基督教、伊斯兰教、道教等等) |  |  |  |  |  |
| 16.我关心下一代 |  |  |  |  |  |
| 17.我的生活很有意义 |  |  |  |  |  |
| 18.总的来说，我满意目前的生活 |  |  |  |  |  |
| 19.在这个世界上，我认为我是有用的 |  |  |  |  |  |
| 20.到这个年龄，我认为我过的和我预想的一样好或比我预想的还要好 |  |  |  |  |  |

**四、社会支持评定量表**

**指导语：以下问题旨在了解您在社会中所获得的支持，请在每小题相应的方框内打“√”。**

1.您有多少关系密切，可以得到支持和帮助的朋友？

□一个也没有 □1－2个 □3－5个 □6个或6个以上

2.近一年来您： （只选一项）

□远离家人，且独居一室

□住处经常变动，多数时间和陌生人住在一起

□和同学、同事或朋友住在一起

□和家人住在一起

3.您和邻居： （只选一项）

□相互之间从不关心，只是点头之交

□遇到困难可能稍微关心

□有些邻居很关心您

□大多数邻居都很关心您

4.您和同事/同学/朋友：（只选一项）

□相互之间从不关心，只是点头之交

□遇到困难可能稍微关心

□有些同事/同学/朋友很关心您

□大多数同事/同学/朋友都很关心您

5.从家庭成员得到的支持和照（在合适的表格内打“对”。）

|  | 得不到支持 | 极少 | 一般 | 全力支持 |
| --- | --- | --- | --- | --- |
| A夫妻（恋人） |  |  |  |  |
| B父母 |  |  |  |  |
| C儿女 |  |  |  |  |
| C兄弟姐妹 |  |  |  |  |
| D其他家庭成员 |  |  |  |  |

6.过去，在您遇到急难情况时，曾经得到的经济支持和解决实际问题的帮助的来源有：

□无任何来源

□有下列来源（可选多项）

□配偶 □其他家人 □亲戚 □朋友 □同事 □工作单位

□党团工会等官方或半官方组织 □宗教、社会团体等非官方组织

□其它（请列出） _________________

7.过去，在您遇到急难情况时，曾经得到的安慰和关心的来源有：

□无任何来源

□下列来源（可选多项）

□配偶 □其他家人 □朋友 □亲戚 □同事 □工作单位

□党团工会等官方或半官方组织 □宗教、社会团体等非官方组织

□其它（请列出） _________________

8.您遇到烦恼时的倾诉方式： （只选一项）

□从不向任何人倾诉 □只向关系极为密切的1-2个人倾诉

□如果朋友主动询问您会说出来 □主动倾诉自己的烦恼，以获得支持和理解

9.您遇到烦恼时的求助方式：（只选一项）

□只靠自己，不接受别人帮助 □很少请求别人帮助

□有时请求别人帮助 □有困难时经常向家人、亲友、组织求援

10.对于团体（如党组织、宗教组织、工会、学生会等）组织活动，您： （只选一项）

□从不参加 □偶尔参加 □经常参加 □主动参加并积极活动

**五、生命意义感量表**

**指导语：以下问题旨在了解您现在的成功老龄化情况，请在每小题后的“从完全没有意义、部分没有意义、有一点意义、中等意义、稍多意义、比较多意义、非常多意义”相应的表格内打“√”。**

| **项目** | **完全没有意义** | **大部分没有意义** | **有一点意义** | **中等意义** | **稍多意义** | **比较多意义** | **非常多意义** |
| --- | --- | --- | --- | --- | --- | --- | --- |
| l我正在寻觅我人生的一个目的或使命。 |  |  |  |  |  |  |  |
| 2我的生活没有明确的目的。 |  |  |  |  |  |  |  |
| 3我正在寻找自己生活的意义。 |  |  |  |  |  |  |  |
| 4我明白自己生活的意义。 |  |  |  |  |  |  |  |
| 5我正在寻觅让我感觉自己生活饶有意义的东西。 |  |  |  |  |  |  |  |
| 6我总在尝试找寻自己生活的目的。 |  |  |  |  |  |  |  |
| 7我的生活有一个清晰的方向。 |  |  |  |  |  |  |  |
| 8我知道什么东西能使自己的生活有意义。 |  |  |  |  |  |  |  |
| 9我已经发现一个让自己满意的生活目的。 |  |  |  |  |  |  |  |
