# Supplementary material for: The impact of social support for older adults in nursing homes on successful aging: a moderated mediation model
Source: Front Public Health. 2024 Feb 21;12:1351953. doi: 10.3389/fpubh.2024.1351953 (PMC10916522; doi:10.3389/fpubh.2024.1351953)
Supplement: Supplementary file 4 [file Table_4.docx]

**The English version of the questionnaires**

This is a paper version of the scale used in the article "The impact of social support for older adults in nursing homes on successful aging: a moderated mediation model" (Manuscript ID: 1351953).We used this questionnaire in our survey of older adults in nursing homes.

Questionnaire on Successful Aging and Related Factors for Older Adults in

Nursing Facilities

Hello, thank you for taking time out of your busy schedule to fill out this questionnaire. We are researchers from Deyang People's Hospital, this is a questionnaire about successful aging of the elderly, this survey is conducted in an anonymous way, the information you answer will be highly confidential and will not be disclosed to others, thank you for your cooperation and support!

1. **General information questionnaire**

**Guidance: The following questions are designed to investigate general information about you, please tick the appropriate box after each sub-question.**

- 1. Your gender ：□Male □Female
  2. Your age：
  3. Level of education:

□ Illiterate □ Elementary school □ Middle school □ High school □ Specialized and above

- 1. Marital status:

□Married □Divorced □Widowed □Unmarried

- 1. Number of children:

□None □1 □2 □3 and above

- 1. Monthly per capita household income:

□ <￥2,000 □ ￥2,000-￥5,000 □ >￥5,000

- 1. Lifestyle
     1. Alcohol consumption:

□ Never drink alcohol □ Occasional drinker □ Regular drinker

- - 1. Smoking:

□ Smoking habit □ No smoking habit or quit smoking

- - 1. Eating

□ Vegetarian diet □ Meat diet □ A reasonable combination of meat and vegetables

- - 1. Exercise :

□ Rarely □ Sometimes □ Often

- - 1. Sleep:

□Normal □Sometimes insomnia □Frequent insomnia

- 1. Chronic Diseases: Please mark all chronic diseases for which you have been diagnosed by a hospital (multiple choice questions).

□High blood pressure Heart disease □Diabetes □Cerebrovascular disease

□ Arthritis □ Cancer Cervical spondylosis Asthma

□ Chronic bronchitis □Emphysema □Peptic ulcer disease □Cataracts

□ Liver cirrhosis □Kidney failure □ Other chronic diseases (if any, please add)

1. **Debilitating index**

**Guidance: The following questions are designed to find out how debilitating it is for you now, please tick the appropriate box for each sub-question.**

**Part I: Functional aspects of the body**

- 1. Do you feel healthy? □Yes □No
  2. Have you lost a lot of weight (6 kg or more in the last 6 months, or 3 kg or more in the last

month, excluding the possibility of weight loss)?

□Yes □No

- 1. Whether the difficulty in walking affects your daily life □Yes □No
  2. Whether difficulties in maintaining balance affect your daily life

□Yes □No

- 1. Does poor hearing affect your daily life? □Yes □No
  2. Does poor vision affect your daily life?

□Yes □No

- 1. Does the lack of strength in your hands affect your daily life? □Yes □No
  2. Does physical fatigue affect your daily life?

□Yes □No

**Part II: Psychological aspects**

- 1. Do you have any memory problems?

□Yes □Sometimes □No

- 1. Have you felt depressed in the last month?

□Yes □Sometimes □No

- 1. Have you felt nervous or anxious in the last month?

□Yes □Sometimes □No

- 1. How well do you handle the problems you encounter?

□Yes □Sometimes □No

**Part III: Social aspects**

- 1. Do you live alone?

□Yes □No

- 1. Would you like to have someone by your side?

□Yes □No

- 1. Can you get enough help from others? □Yes □No

1. **Questionnaire on successful ageing**

**GUIDELINE: The following questions are designed to find out how successfully you are ageing now, please tick the appropriate table for "Never, Occasionally, Sometimes, Often, Always" after each sub-question.**

| sports event | never | infrequent | now and  then | non- recurrent | always |
| --- | --- | --- | --- | --- | --- |
| 1. I can take care of things that need to be  taken care of at home and take care of myself (eating, bathing, dressing, etc.) |  |  |  |  |  |
| 2. So far, I can cope with the physical  changes that come with aging |  |  |  |  |  |

| 3. I am optimistic about my future life |  |  |  |  |  |
| --- | --- | --- | --- | --- | --- |
| 4. I feel I can cope with getting older |  |  |  |  |  |
| 5. I feel I can handle life's problems |  |  |  |  |  |
| 6. I can think of a solution to the problem |  |  |  |  |  |
| 7. I am good at thinking of new ways to  solve problems |  |  |  |  |  |
| 8. I like to try new things |  |  |  |  |  |
| 9. I am optimistic and cheerful |  |  |  |  |  |
| 10. I miss my loved ones who have passed  away and feel close to them. |  |  |  |  |  |
| 11. I spend some time praying or attending  some religious services |  |  |  |  |  |
| 12. the way I think about the world has  changed as I've gotten older |  |  |  |  |  |
| 13. I would like to have a few close friends  rather than a lot of regular friends |  |  |  |  |  |
| 14. There are sometimes two correct answers  to a question or situation |  |  |  |  |  |
| 15. There is a faith that is very important to me (e.g., Communism, Buddhism,  Christianity, Islam, Taoism, etc.) |  |  |  |  |  |
| 16. I care about the next generation |  |  |  |  |  |
| 17. My life is meaningful. |  |  |  |  |  |
| 18. In general, I am satisfied with my current  life |  |  |  |  |  |
| 19. In this world, I think I'm useful |  |  |  |  |  |
| 20. At this age, I think I'm doing as well or  better than I expected |  |  |  |  |  |

1. **Social Support Rating Scale**

**Guidance: The following questions are designed to find out about the support you receive in the community, please tick the appropriate box for each sub-question.**

- 1. How many close friends do you have who could use support and help?

□ none □ 1-2 □ 3-5 □ 6 or more

- 1. In the past year you: (check one only)
- Far from family and alone in a room
- Frequent changes of residence, most of the time with strangers
- Living with classmates, colleagues or friends
- Living with family
  1. You and your neighbors: (check one only)
- Never cared about each other, just nodded at each other
- In trouble, I might be a little bit concerned
- Some of your neighbors care about you .
- Most of the neighbors care about you
  1. You and coworkers/classmates/friends: (check one only)
- Never cared about each other, just nodded at each other
- In trouble, I might be a little bit concerned
- Some of your coworkers/classmates/friends care about you
- Most coworkers/classmates/friends care about you
  1. Support and care received from family members (check "yes" in the appropriate table).

|  | No support. | minimal | general | full support |
| --- | --- | --- | --- | --- |
| A.Husband and  wife (lovers) |  |  |  |  |
| B.spouse |  |  |  |  |
| C.sons and  daughters |  |  |  |  |
| C.siblings |  |  |  |  |
| D.Other family  members |  |  |  |  |

- 1. In the past, the sources of financial support and help in solving practical problems that you have received in times of emergency are:
- Without any source
- There are the following sources (you may choose more than one)

□spouse □other family member □relative □friend □coworker □workplace

- Official or semi-official organizations such as political parties and trade unions □

Unofficial organizations such as religious and social groups

- Other (please list)
  1. In the past, the sources of comfort and concern you have received in times of emergency have been:
- Without any source
- The following sources (check more than one)

□spouse □other family member □friend □relative □coworker □workplace

- Official or semi-official organizations such as political parties and trade unions □

Unofficial organizations such as religious and social groups

- Other (please list)
  1. How do you talk about your troubles: (check one only)

□ Never confides in anyone □ Confides only in 1-2 people who are very close to him/her

□ You will speak up if a friend asks □ Take the initiative to talk about your worries to get support and understanding

- 1. How do you seek help when you are troubled: (check one only)

□ rely only on themselves and do not accept help from others □ seldom ask for help from others

□ Sometimes ask for help from others □ Often ask for help from family, relatives, friends, or organizations when in trouble

- 1. Regarding the activities organized by groups (e.g., party organizations, religious organizations, trade unions, student unions, etc.), do you: (check one only)

□ Never participated □ Occasionally participated □ Regularly participated □ Active

and active

1. **Sense of Meaning of Life Scale**

**GUIDELINE: The following questions are designed to find out how successfully you are aging now, so please tick the appropriate table "from not at all meaningful, partially meaningful, a little meaningful, moderately meaningful, a little more meaningful, a little more meaningful, more meaningful, and very much meaningful" after each sub-question.**

| **sports event** | **It doesn't make any sense**  **at all.** | **Most of it**  **doesn't make sense.** | **Makes a little sense.** | **of moderate significance** | **make slightly more sense** | **make more sense** | **make perfect sense** |
| --- | --- | --- | --- | --- | --- | --- | --- |
| l. I am seeking a purpose or mission in  my life. |  |  |  |  |  |  |  |
| 2. My life has no clear  purpose. |  |  |  |  |  |  |  |
| 3. I am searching for  meaning in my life. |  |  |  |  |  |  |  |
| 4. I understand the  meaning of my life. |  |  |  |  |  |  |  |
| 5. I'm on a quest to find something that makes me feel like I'm living a meaningful life. |  |  |  |  |  |  |  |
| 6. I'm always trying to find my purpose in life. |  |  |  |  |  |  |  |
| 7. I have a clear  direction in my life. |  |  |  |  |  |  |  |
| 8 I know what makes  sense of my life. |  |  |  |  |  |  |  |
| 9. I have found a purpose in life that  satisfies me. |  |  |  |  |  |  |  |
